# Particle contact evolution: A precursor mechanism for earthquake-induced liquefaction prior to pore-pressure buildup

**DOI:** 10.1016/j.xinn.2026.101394

**Published:** 2026-04-09

**Authors:** Hu Zheng, Yuxiang Hu, Yiqiu Zhao, Joshua A. Dijksman, Minyi Zhu, Dong Wang, Wuwei Mao, Wei Teng, Lihui Li, Yufeng Gao, Yu Huang

**Affiliations:** 1 Department of Geotechnical and Hydraulic Engineering, College of Civil Engineering, Tongji University, Shanghai 200092, China; 2 State Key Laboratory of Disaster Reduction in Civil Engineering, Tongji University, Shanghai 200092, China; 3 Department of Physics, The Hong Kong University of Science and Technology, Hong Kong SAR 999077, China; 4 Van der Waals-Zeeman Institute, Institute of Physics, University of Amsterdam, Amsterdam 1098 XH, the Netherlands; 5 Department of Mechanical Engineering, Yale University, New Haven, CT 06511, USA; 6 State Key Laboratory for Pollution Control, School of Environmental Science and Engineering, Tongji University, Shanghai 200092, China; 7 State Key Laboratory of Lithospheric and Environmental Coevolution, Institute of Geology and Geophysics, Chinese Academy of Sciences, Beijing 100029, China; 8 Key Laboratory of Ministry of Education for Geomechanics and Embankment Engineering, Hohai University, Nanjing 210024, China

Dear Editor,

Earthquake-induced soil liquefaction is a destructive geohazard that repeatedly damages buildings, lifelines, and critical infrastructure during strong shaking. It has traditionally been attributed to cyclic loading that elevates pore-water pressure, reduces effective stress, and destabilizes the load-bearing particle skeleton.^1^ Previous works^2^^,^^3^ have suggested that liquefaction is closely associated with changes in the internal particle structure, particularly the breakdown of interparticle contact networks. However, these insights are often based on indirect observations or numerical assumptions and lack direct experimental validation of the underlying mechanism.

In this letter, we establish an experimentally decoupled validation framework for investigating the sequence of microstructural and hydraulic events in earthquake-induced liquefaction of saturated loose sand. Building on important insights from recent discrete-element simulations,^2^^,^^3^ and recognizing that grain-scale physical experiments that resolve contact-network evolution and hydraulic response remain relatively scarce, we combine complementary physical experiments with simplified numerical modeling. This framework enables systematic examination of the interplay among contact degradation, contractive volumetric change, and excess pore-pressure development. Specifically, computed tomography (CT) reconstruction with three-dimensional (3D) printing isolates hydraulic effects, photoelastic measurements capture real-time interparticle force evolution, and computational fluid dynamics-discrete element method‌ (CFD-DEM) modeling probes post-instability fluid dynamics. Together, these approaches provide a particle-scale route for studying liquefaction, with potential for future integration into unified 3D measurements of contact evolution and pore pressure.

We first designed an idealized limiting-case experiment in which contact evolution is mechanically suppressed while the pore geometry is kept fixed so that the hydraulic response can be isolated. Although this setup cannot fully reproduce the complete behavior of natural sand, it provides a controlled way to isolate the hydraulic response from structural evolution. A loose quartz-sand specimen was scanned by X-ray CT, and the 3D pore architecture was reconstructed at a representative-elementary-volume (REV) scale (800 × 800 × 800 pixels). This REV was then used to compare and analyze the microstructures of the natural sand and the 3D printed replica. Using stereolithography, we printed a rigid, monolithic replica that preserved the specimen’s overall dimensions (180 mm in diameter and 100 mm in height), porosity, coordination number, and tortuous flow paths but mechanically bonded the grains into a continuous solid (Figures 1A–1G). We then conducted one-degree-of-freedom horizontal shaking tests on both saturated natural sand (Figure 1H) and the saturated printed analog (Figure 1I) under identical boundary conditions. The horizontal displacement amplitude was fixed at 5 mm. For the natural quartz-sand specimen tested in this study, no liquefaction and no measurable excess pore-pressure rise were observed at 4 Hz. When the frequency reached 5 Hz and above, liquefaction was consistently triggered within a few loading cycles, as evidenced by a rapid increase in excess pore pressure followed by gradual dissipation toward the initial level (Figure 1J). In sharp contrast, under the same loading history and identical hydraulic boundary conditions, the saturated 3D-printed specimen exhibited no significant positive excess pore-pressure buildup across the tested frequency range, with the pore-pressure signal remaining close to the initial baseline throughout the excitation (Figure 1K). Because contact rearrangement is precluded in the printed material, these observations demonstrate that cyclic loading does not generate excess pore pressure in the absence of contact failure. In the natural sand, cyclic shear leads to contact loss and irreversible contractive compaction. As the pore volume decreases, the nearly incompressible pore fluid resists volume change and an excess pore pressure is generated.^6^ In the shaking-table liquefaction tests on saturated loose sand conducted in this study, the excess pore pressure observed in liquefying sand therefore accompanies, rather than precedes, the structural collapse of the contact network.

**Figure 1:**
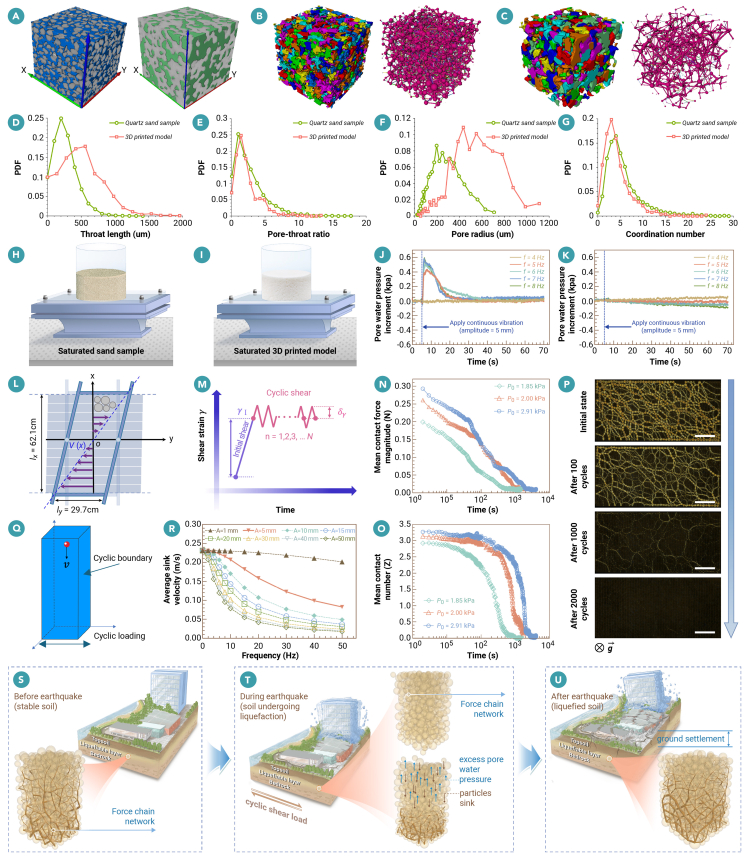
Contact-first mechanism of liquefaction (A) Spatial distribution of pores in the quartz-sand model (left) and 3D-printed model (right). (B) Pore network of the quartz-sand model. Left: different colors represent different pore clusters, which are collections of connected pores. Right: the slender rod-like segments represent throats, and the spherical segments represent pores. (C) Pore network of the 3D printed model. (D) Probability density function (PDF) of throat length within the two samples. (E) PDF of pore-throat ratio within the two samples. (F) PDF of pore radius within the two samples. (G) PDF of coordination number within the two samples. (H) Shake-table test of saturated sand. (I) Shake-table test of saturated 3D printed model. (J) Evolution patterns of pore-water pressure in saturated quartz sand under cyclic loading. (K) Evolution patterns of pore-water pressure in saturated 3D-printed model under cyclic loading. (L) A top view schematic of the multi-slat shear cell. (M) The driving-strain protocol. The experimental setup and procedures follow those described in Zhao et al.^4^ (N) The evolution of mean contact-force magnitude. (O) The evolution of mean contact number for the same experiments shown in (N). (P) The top view of the horizontal two-dimensional model granular system imaged through polarizers. (Q) Model setup of a single spherical particle settling in a water tank. The implementation details follow those described by Wang et al.^5^ (R) The average sink velocity of a single particle in water from rest over a distance of 10 cm. (S) Shear-jamming state with a weak force-chain network. (T) Unjammed state with no force-chain network and particles sink and form new force chains. (U) Jammed state with a strong force-chain network.

To demonstrate that cyclic loading in the absence of pore-pressure effects can by itself weaken the load-bearing contact network, and to obtain high temporal resolution measurements of the evolution of interparticle contact forces under cyclic loading, displacement-controlled quasi-static cyclic shear experiments were carried out on a horizontal, gravity-free, bi-disperse photoelastic disk assembly (Figure 1L). In a typical experiment, the model granular sample was confined in a multi-slat simple shear cell and formed a packing fraction of ∅ = 0.816. This packing fraction falls in the range between the random loose-packing fraction^7^ and the random close-packing fraction^8^ in two dimensions. For each experiment, the initial state was prepared by imposing a quasi-static homogeneous shear strain *γ*_*I*_ to a stress-free state (Figure 1M). The shear was applied step by step and the particles were allowed to relax between steps, resulting in a quasi-static process. This imposed shear drives the system into “jammed” states that feature anisotropic force networks and statically support non-zero shear stress and so mimics the state of many natural soils that have an extended shear history.^4^ Increasing *γ*_*I*_ leads to a larger pressure *p*_0_ in the jammed state. To initiate liquefaction, the jammed states were then subjected to a series of cyclic shear with a strain amplitude *δ*_*γ*_ above the liquefaction threshold ${\delta_{\gamma }}^{*}$ (Figure 1M).

Figure 1N plots the evolution of the measured mean contact-force magnitude ⟨|*f*_*i*_|⟩ for states formed after each complete shear cycle for three example experiments with different initial pressure *p*_0_ = 1.85, 2.00, and 2.95 kPa. For each test, the shear-strain amplitude was fixed at 0.95%. Clearly, when the number of shear cycles reached about 1,000, ⟨|*f*_*i*_|⟩ decreased to near zero, and when the number of cycles reached approximately 2,000, ⟨|*f*_*i*_|⟩ effectively vanished, which corresponds to the state of zero effective stress during the liquefaction. Moreover, a lower *p*_0_ led to a more rapid loss of interparticle contact forces. To quantify the structural change, we measured the mean contact number ⟨*z*⟩≡2*N*_*c*_/*N*_*p*_, where *N*_*c*_ is the total number of force-bearing contacts and *N*_*p*_ is the total number of particles. As shown in Figure 1O, ⟨*z*⟩ drops to zero as ⟨|*f*_*i*_|⟩ vanishes, thus providing direct evidence for the collapse of the contact structure. This process can also be clearly seen directly from the photoelastic images shown in Figure 1P. The force chains captured by the photoelastic patterns from images obtained with polarizers clearly disappear after about 2,000 shear cycles. While our experiments were conducted in a horizontal plane, we expect that the collapse dynamics of the particle network remain qualitatively the same when gravity presents. However, the strain amplitude needed to initiate liquefaction may differ due to the additional pressure exerted by the gravitational force.^4^

This set of experiments demonstrates that, even in the absence of a water, dry granular materials in a shear-jammed state will lose their rigidity under small-amplitude quasi-static cyclic shear. Under the specific conditions investigated in this study, namely saturated loose sands subjected to earthquake-like cyclic shear under laboratory drainage conditions, the results support the view that excess pore-water pressure is not the primary trigger of liquefaction. Instead, the dominant mechanism under the tested conditions is the progressive loss of effective load-bearing contacts between soil particles during cyclic loading.

Cyclic shear-stress variation disrupts the soil particle packing, causing grains to lose effective contacts and become temporarily suspended. Under gravity, these particles then sink and gradually re-establish effective contacts, forming a new force-bearing configuration. Once the skeleton has collapsed, the recovery process is primarily governed by the settling behavior of individual particles in the fluid and the associated reformation of force-bearing contacts. To isolate this hydrodynamic contribution, a one way coupled CFD-DEM simulation^5^ of a single sphere settling in an oscillatory liquid flow is employed (Figure 1Q), which captures the competition between gravity and unsteady fluid drag without additional assumptions on packing fabric or interparticle contacts. The results show that cyclic fluid motion significantly slows down particle settling (Figure 1R), indicating that water can substantially prolong the time needed to rebuild the force network after liquefaction and thereby increase the persistence and severity of liquefaction. While this analysis focuses on settling velocity as a first-order descriptor, recent DEM^9^ and SPH-DEM^10^ studies have shown that additional fabric measures, such as mean neighboring particle distance, coordination and force anisotropy, and directional fabric tensors capture important aspects of post-liquefaction shear deformation and structural recovery. Building on the theoretical framework established in this work, future studies will combine the present experimental approach with detailed numerical modeling to extract these richer fabric descriptors and to enable a more comprehensive quantitative comparison with existing micro-mechanical studies of liquefaction and recovery.

The three strands of evidence combine into a coherent particle-scale interpretation of earthquake-induced liquefaction in saturated loose sand under the laboratory drainage conditions considered here. Prior to shaking, a loose, contractive sand resides in a metastable, shear-jammed state supported by fragile, anisotropic force-chain networks (Figure 1S). Earthquake-like cyclic shear perturbs this network; once the imposed strain amplitude exceeds a pressure-dependent threshold, contacts fail collectively, the mean contact number rapidly drops, and the effective stress carried by the skeleton collapses (Figure 1T). As gravitational compaction proceeds, pore volume decreases and an excess pore pressure is generated in the nearly incompressible pore fluid, which further reduces the effective stress transmitted at contacts (Figure 1T). Because fluid viscosity and unsteady hydrodynamics slow the settling of particles and the rebuilding of force chains, water prolongs the fluid-like response. Eventually, as compaction completes and new contacts lock, the assembly re-enters a jammed state with restored rigidity (Figure 1U).

It is worth noting that this study aims to extract a general and transferable physical mechanism. Accordingly, a representative granular material (quartz sand) and simplified cyclic loading are adopted without systematically varying factors such as particle shape, grading, initial fabric, or loading complexity. These simplifications reflect the focus on microstructural evolution after instability. While such factors influence the onset of liquefaction, the fundamental particle-scale mechanism governing post-instability behavior is expected to remain unchanged. Future work will systematically examine their effects on liquefaction resistance and contact-network evolution.

In summary, for the regime of earthquake-induced liquefaction of saturated loose sands investigated in this letter, the present results support a sequence in which contact-network collapse precedes excess pore-pressure buildup under the specific regime investigated. Excess pore pressure is generated during subsequent compaction and amplifies deformation but is not the primary trigger of failure. This sequence is not universal; in other scenarios, such as seepage-induced liquefaction, pore pressure may develop earlier and interact bidirectionally with contact degradation. Despite additional complexities in field conditions, the framework developed here provides a particle-scale basis for understanding earthquake-induced liquefaction and can be extended to more realistic systems.

## Funding and acknowledgments

This work was supported in part by the 10.13039/501100001809National Natural Science Foundation of China (grant nos. 42277156 and 42120104008). The photoelastic experiments were carried out at Duke University under the support of NSF DMR 1809762. We would like to express our sincere gratitude to Joshua E.S. Socolar for his invaluable support and contributions throughout this work. We are also deeply thankful to Ikuo Towhata from Japan, whose insights and expertise have greatly enriched our research, and to Hehua Zhu and Feng Zhang for their guidance and assistance. We additionally thank Ryan Hurley for his helpful discussions and encouragement during the development of this study.

## Declaration of interests

The author declare no competing interests.
